# Validation and Trustworthiness of Multiscale Models of Cardiac Electrophysiology

**DOI:** 10.3389/fphys.2018.00106

**Published:** 2018-02-15

**Authors:** Pras Pathmanathan, Richard A. Gray

**Affiliations:** Office of Science and Engineering Laboratories, Center for Devices and Radiological Health, U.S. Food and Drug Administration, Silver Spring, MD, United States

**Keywords:** credibility, calibration, validation, computational modeling, cardiac models

## Abstract

Computational models of cardiac electrophysiology have a long history in basic science applications and device design and evaluation, but have significant potential for clinical applications in all areas of cardiovascular medicine, including functional imaging and mapping, drug safety evaluation, disease diagnosis, patient selection, and therapy optimisation or personalisation. For all stakeholders to be confident in model-based clinical decisions, cardiac electrophysiological (CEP) models must be demonstrated to be trustworthy and reliable. Credibility, that is, the belief in the predictive capability, of a computational model is primarily established by performing validation, in which model predictions are compared to experimental or clinical data. However, there are numerous challenges to performing validation for highly complex multi-scale physiological models such as CEP models. As a result, credibility of CEP model predictions is usually founded upon a wide range of distinct factors, including various types of validation results, underlying theory, evidence supporting model assumptions, evidence from model calibration, all at a variety of scales from ion channel to cell to organ. Consequently, it is often unclear, or a matter for debate, the extent to which a CEP model can be trusted for a given application. The aim of this article is to clarify potential rationale for the trustworthiness of CEP models by reviewing evidence that has been (or could be) presented to support their credibility. We specifically address the complexity and multi-scale nature of CEP models which makes traditional model evaluation difficult. In addition, we make explicit some of the credibility justification that we believe is implicitly embedded in the CEP modeling literature. Overall, we provide a fresh perspective to CEP model credibility, and build a depiction and categorisation of the wide-ranging body of credibility evidence for CEP models. This paper also represents a step toward the extension of model evaluation methodologies that are currently being developed by the medical device community, to physiological models.

## Introduction

One of the most remarkable properties of the natural world is that is it can be understood using mathematical equations—a property described by Eugene Wigner as “the unreasonable effectiveness of mathematics in the natural sciences.” Once the appropriate mathematical groundwork had been developed, it became possible to describe intricate and multi-faceted physical phenomena using relatively simple mathematical equations, e.g., fluid flow, deformation of solid bodies, electromagnetic wave propagation, and phenomena at widely different scales from atoms to galaxies. Computational models, which are mathematical models solved by means of a computer, can be used to solve governing equations underlying complex systems and simulate their behavior. The remarkable predictive capability of computational models based on the fundamental laws of physics has enabled such models to be routinely used in a multitude of engineering applications.

Biology, in contrast to physics, is less easily characterized by simple or small numbers of mathematical equations. Primarily, this is due to the complexity and variability in biological processes which makes them inherently non-linear, multi-disciplinary and multi-scale. While computational models of human physiological processes have been developed and refined for decades, they are not as predictive as computational models in engineering, and likely never will be. Nevertheless, biomedical computational models have without doubt the potential for revolutionizing medicine just as physics-based models have forever changed research, design, and evaluation in engineering.

**Figure 1:**
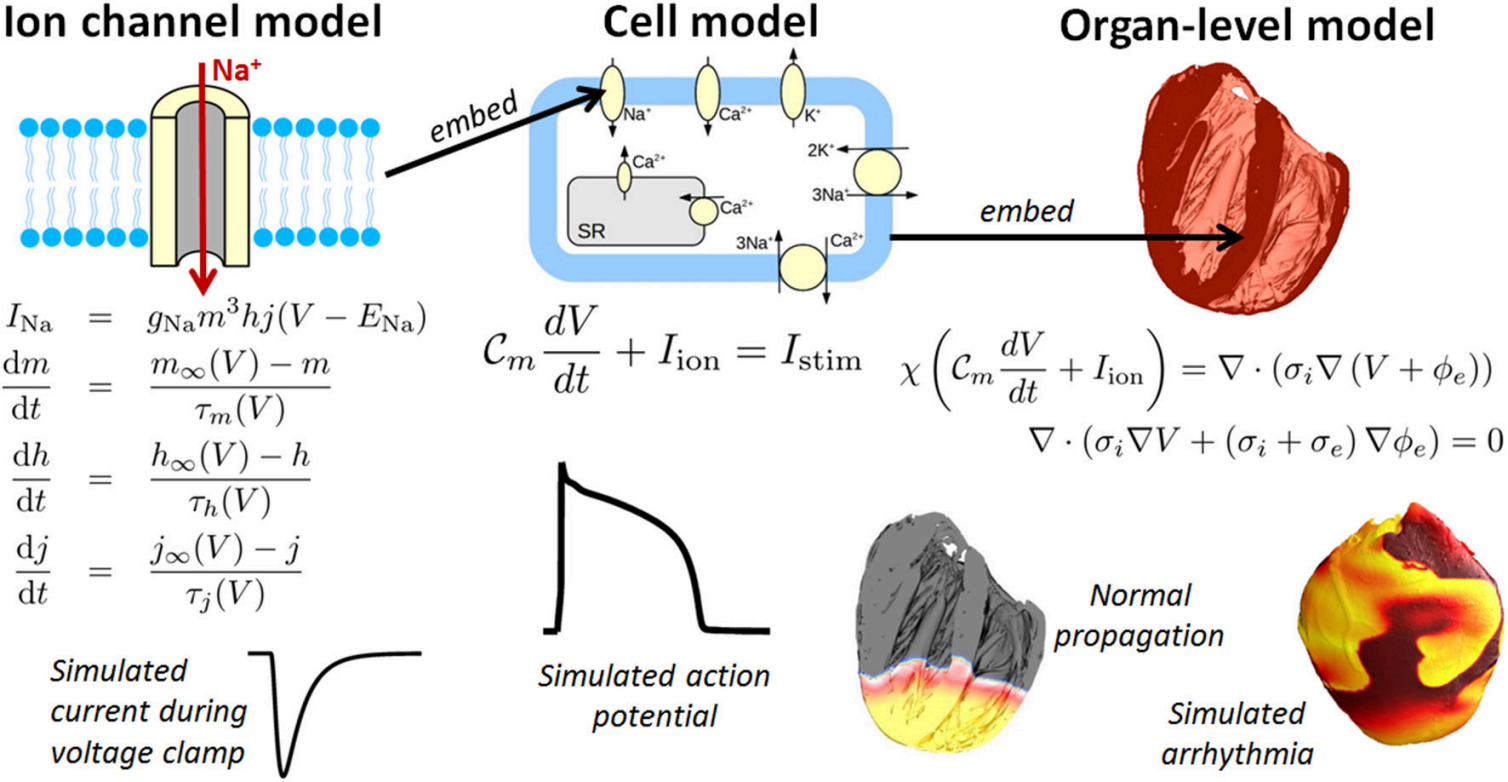
Components of a multiscale cardiac electrophysiology (CEP) model. **(Left)**: equations and sample output for a Hodgkin-Huxley formulation of the rapid sodium current. Multiple such sub-cellular models can be used to define a cell model. **(Center)**: schematic of sub-cellular processes included in a hypothetical cell model, together with the differential equation governing the transmembrane voltage, and sample output. Cell models differ in their formulation of the ionic current I_ion_ and can be made up of dozens of ordinary differential equations. **(Right)**: Cell models can be incorporated into the bidomain equations and solved on a computational mesh of the heart [top right: high-resolution rabbit biventricular mesh of Bishop et al. (2010)], to simulate normal or arrhythmic cardiac activity (bottom right).

One field which holds considerable promise for clinical applications is cardiac modeling, owing to the maturity of the field (Trayanova, 2011) (Winslow et al., 2012) and the fact that heart disease is the leading cause of the death in the industrialized world. Computational cardiac models can simulate the electrophysiology and/or mechanical deformation of cardiac myocytes, tissue, or the whole heart. This paper is focused on cardiac electrophysiological (CEP) models. Figure 1 illustrates the typical components to a CEP model, which are usually multi-scale, containing as a fundamental unit a cellular model of myocyte EP activity. Such “cell models,” of which over a 100 have been published of varying complexity and for a range of mammalian species, are typically sets of ordinary differential equations (ODEs), and predict the action potential (AP) and many other cellular and sub-cellular quantities. Notable recent human cell models include (Iyer et al., 2004; ten Tusscher et al., 2004; ten Tusscher and Panfilov, 2006; Grandi et al., 2010) and (O'Hara et al., 2011). For reviews of single cell models, see (Fink et al., 2011; Noble, 2011; Noble et al., 2012). Cell models are often composed of multiple sub-models, for different ion channels, pumps and exchangers or representing subcellular processes such as calcium handling. These sub-models are usually also systems of ODEs. To simulate electrical wave propagation, including arrhythmic activity, in tissue or the whole heart, cell models are coupled to partial differential equations (PDEs) known as the “bidomain” or “monodomain” equations (Clayton et al., 2011; Franzone et al., 2014; Lopez-Perez et al., 2015); see Figure 1. A further extension is to model the heart embedded in the torso, which allows for simulation of the electrocardiogram (ECG) (Richards et al., 2013; Zemzemi et al., 2013). In recent years various imaging, modeling and computational advances have enabled *patient-specific* heart models to be generated using clinical data (see e.g., Smith et al., 2011; Chabiniok et al., 2016 for discussions). Anatomical personalisation using clinical imaging data is most common (e.g., Arevalo et al., 2016), although personalisation of functional (Chen et al., 2016) or material (Aguado-Sierra et al., 2011) parameters using clinical data has also been performed. Patient-specific models can be used to make patient-specific clinical predictions and represent an important step forward toward precision medicine.

All types of CEP model—ion channel or subcellular models, cell models, tissue, and organ-level models—have proved to be powerful tools complementing experiment in basic cardiac electrophysiological research (Plank et al., 2009), for understanding mechanisms behind both normal rhythm and cardiac arrhythmias. However, CEP models also have potential applications in all aspects of cardiovascular medicine, including device design and evaluation, functional imaging and mapping, drug safety evaluation, disease diagnosis, patient selection, and therapy optimisation or personalisation. There are numerous reviews covering the current and potential applications of CEP models; (see e.g., Trayanova and Boyle, 2014; Davies et al., 2016; Niederer and Smith, 2016). However, one aspect of the modeling which has been inadequately covered in the current cardiac modeling literature is rationale for when and why cardiac models can be *trusted*.

The **credibility** of a computational model has been defined as the belief in the predictive capability of the model for a given intended use (ASME, 2017) or the willingness of people to trust a model's predictions (Patterson and Whelan, 2017). Typically, credibility of a computational model is founded upon **validation** results. Validation is the process of testing a model by comparing model predictions to experimental or clinical data. (A more precise definition is provided below). However, other types of evidence (non-validation evidence) can also be used to argue that a model is sufficiently credible for its intended use. As we explain below, the credibility of CEP models is typically founded upon a wide range of factors, and consequently it can be very unclear, or a matter for debate, the extent to which a cardiac model can be trusted for a given application. In fact, many papers in the literature leave implicit why, and to what extent, the model predictions can be considered credible.

The aim of this article is to clarify and discuss reasons that could support the trustworthiness of CEP models. We will do so by reviewing different types of evidence that have been presented to support CEP model credibility, or are otherwise relevant to the assessment of credibility, hereafter referred to as CEP model **credibility evidence**. The review will include: (i) discussion of common practice regarding CEP model validation; (ii) examples of strategies taken for performing CEP model validation; and (iii) discussion of other credibility evidence for CEP models, including historical evidence that often *implicitly* supports simulation studies. The review will *not* aim to evaluate specific cardiac models or in any way judge the *quality* of any validation results or other evidence. Such decisions require difficult judgements based on careful consideration of all available evidence, in the context of the precise application that model is to be used for (including assessment of the risks associated with inaccurate predictions NASA, 2009) and are therefore beyond the scope of this review. In other words, we are *not* claiming or implying that any CEP model “is” or “is not trustworthy”; nor do we argue that any modeling approach or process is or is not trustworthy. Instead, our focus will be on *types* of evidence that could, in principle, support the trustworthiness of a model for a given intended use.

In previous work we advocated that engineering model assessment approaches known as verification, validation, and uncertainty quantification (VVUQ) could be important in the advancement of cardiac CEP modeling (Pathmanathan and Gray, 2013) and explored verification (Pathmanathan and Gray, 2014) and uncertainty quantification (UQ) for CEP models (Pathmanathan et al., 2015). This paper continues this line of work by focusing on validation-related activities. Only activities related to comparison of the model to the real world are within the scope of this review. Therefore, activities such as code verification, calculation verification, and sensitivity analysis, while important for overall assessment of credibility and receiving increasing interest in the field (Sobie, 2009; Niederer et al., 2011; Chang et al., 2015) are outside the scope of this paper. Additionally, while uncertainty quantification is related to validation as will be described in section Why Trust a Computational Model?, research on the process of performing uncertainty quantification with CEP models is also outside the scope of the review, though this is also receiving increasing recent interest in CEP; (see e.g., Konukoglu et al., 2011; Johnstone et al., 2016; Chang et al., 2017).

In fact, there is enormous current interest across computational science in methods and best practices for demonstrating or evaluating the reliability of computational models (National Research Council, 2012). The medical device community is collaborating on a Standard for assessing credibility of computational models for medical device applications (ASME, 2017). However, this Standard and related medical device Guidances (Food and Drug Administration, 2016) are motivated by traditional “physics-based” engineering models in biomedical applications [e.g., models based on solid mechanics (Pelton et al., 2008), fluid dynamics (Stewart et al., 2012), or electromagnetism (Angelone et al., 2010)]. The relevance of such approaches to the evaluation of complex physiological models such as CEP models is limited. In particular, while both (Food and Drug Administration, 2016) and (ASME, 2017) address validation, they do not account for the possibility of multiple sources of different types of validation evidence, or other evidence which could support credibility. In this paper, we demonstrate how a large body of evidence will generally support a CEP model. By exposing and discussing this wide range of potential credibility evidence for CEP models, this paper is a necessary first step toward the extension of the above approaches to cardiac and other physiological models.

The paper is organized as follows. In section Why Trust a Computational Model? we categorize and discuss different types of credibility evidence, and discuss validation in detail. Section Credibility of CEP Models at Different Spatial Scales then reviews credibility evidence for CEP models organized by spatial scale. Section Discussion summarizes and discusses our conclusions.

## Why trust a computational model?

**Figure 2:**
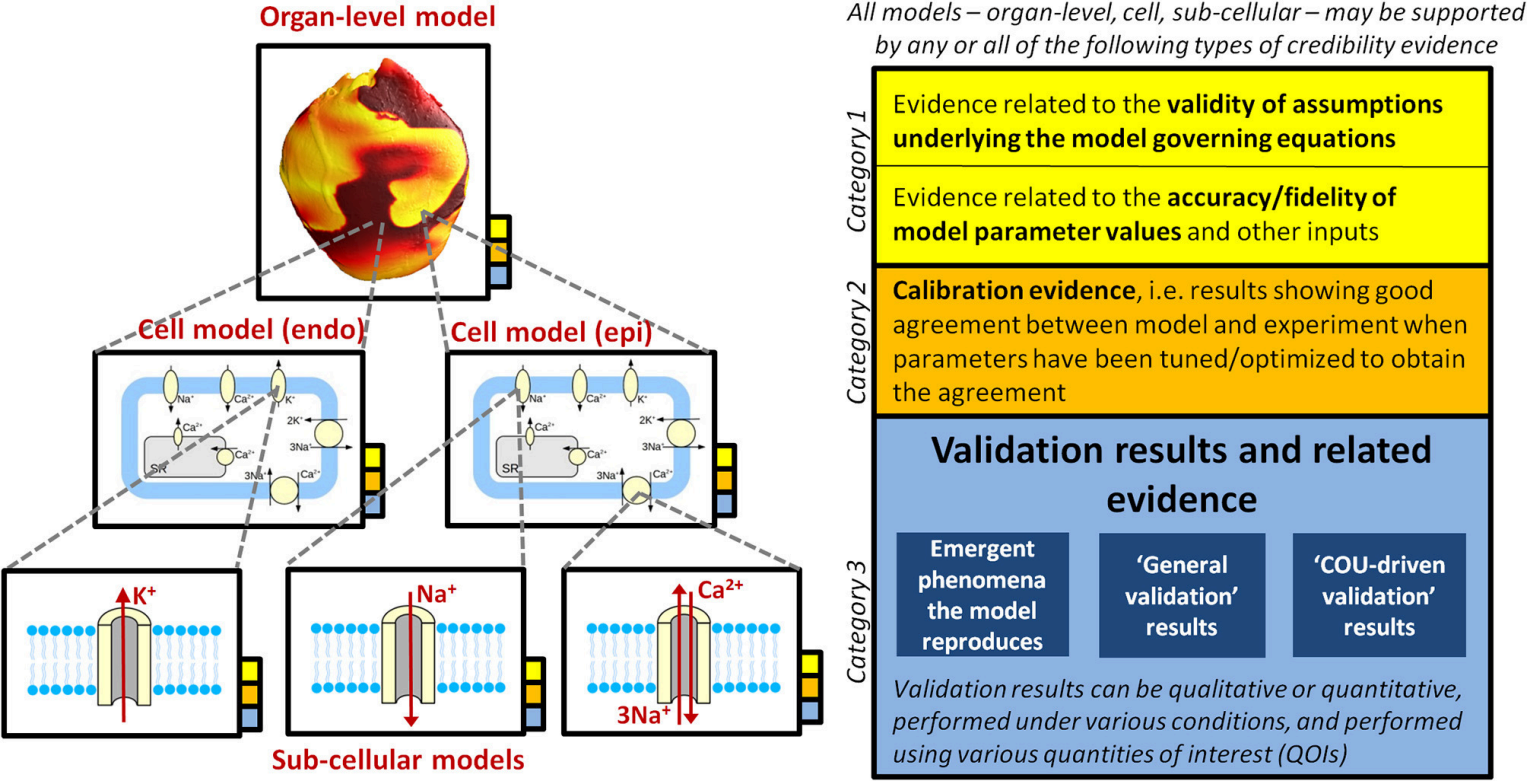
Illustration of how a multiscale CEP model may be supported by multiple sources of credibility evidence (that is, evidence relevant when assessing the credibility of the model), taken from model evaluation at multiple scales. The overall model (i.e., organ-level model), the underlying cell models (here it is assumed that the organ-level model incorporates two different cell models, one for epicardial tissue, one for endocardial), and all underlying sub-cellular models may all be supported by the different types of evidence presented in the right of the figure. See section Why Trust a Computational Model? for full discussion.

Figure 2 provides an overview of the concepts discussed throughout this section. Various types of rationale could be used to argue for the credibility of a computational model. The following are three distinct categories of evidence that could support *some* level of confidence in a model:

*Category 1:* Evidence related to the **validity of assumptions underlying the model governing equations**, together with evidence related to the **accuracy/fidelity of model parameter values** and other inputs. These are grouped together because if the equations are considered appropriate, but there is no confidence that the parameters are accurate, then there will be little confidence in model predictions, and vice versa.

*Category 2:* **Calibration evidence**. Calibration is the process of tuning, fitting or optimizing parameters in a model so that the model results match experimental or clinical data. Calibration is primarily performed to determine model parameter values that cannot be directly measured. However, if the results from calibration demonstrate a good match between model and the experimental/clinical data, the results could potentially *also* be used as evidence for credibility of the model.

*Category 3:* Evidence generated from testing the predictive capability of the *completed* model. This includes **validation evidence**, that is, comparison of model predictions to independent real-world data *not used* in the construction of the model (Roache, 2009). Validation is discussed in detail later in this section.

These are distinct types of evidence and may provide very different levels of confidence in a model. The first category is based on model equations, assumptions and parameter values, but not on actual model outputs or results, with no actual testing of the model. This category includes historical evidence supporting the governing equations that were used in the model. It also includes evidence regarding the quality of data used to determine model parameters. The second category is based on comparing model outputs with experimental/clinical data, but allows for model parameters to be altered for the model to match the data. Calibration results are regularly used, either implicitly and explicitly, as evidence for credibility of biological computational models. If a model's parameters can be chosen so that the model reproduces certain experimental data, this ability to fit the data or to reproduce phenomena could be used in support of the model—especially when a complex phenomenon is reproduced using a model with only a few parameters. The last category—validation and related evidence—is obviously the strongest test of the model: it assumes the model has been completely defined and *then* its ability to reproduce the real world is tested.

For many applications—in particular the basic science applications of hypothesis generation and mechanistic insight—use of a model that has no supporting validation evidence may be perfectly appropriate. Mathematical models in biology can be thought of a means in which existing knowledge or hypotheses are integrated (Brodland, 2015), in which case running a simulation is simply an efficient means of determining the logical consequences of those knowledge/hypotheses, impossible through mental deduction alone for complex systems. This is one of the reasons why mathematical modeling has proven a successful complement to experiments in understanding biological processes (Noble, 2011). However, when a model is to be used in decision-making, and in particular for high-risk applications such as safety-critical clinical applications, validation becomes very important. (Carusi et al., 2012) provide a discussion on the meaning of CEP models as representations. Patterson and Whelan (2017) provide an excellent general discussion on models as representations vs. as predictive tools, and propose a high-level framework for deciding how to evaluate models along this spectrum.

### Validation

Validation has been described as “*the assessment of the accuracy of a computational simulation by comparison with experimental data*” (Oberkampf et al., 2004). One definition initially proposed by the Department of Defense (DoD) and commonly used by the engineering community and elsewhere (Oberkampf et al., 2004; Roache, 2009; National Research Council, 2012), including increasingly within the medical devices community (Food and Drug Administration, 2016), (ASME, 2017), is: “*the process of determining the degree to which a model is an accurate representation of the real world from the perspective of the intended uses of the model*.” This definition emphasizes that the validation process is dependent on the specific “intended use” of the model, also referred to as the “model application” or the “**context of use**” **(COU)**. (Roache, 2009) provide a good discussion of the DoD definition of validation, and explains how many different interpretations of it are made even within the engineering community. Moreover, there is no inter-disciplinary consensus on a precise definition of validation, and different communities may have very different understandings of what constitutes model validation. (Bellocchi et al., 2010) list 20 definitions of model validation proposed between 1960 and 2010. (Eddy et al., 2012) describe a categorisation of validation used in the health economics and outcomes community. (Patterson and Whelan, 2017) describe a broad concept of validation of biological models, which includes but is more expansive than the engineering/DoD understanding of validation. (Viceconti, 2011) refer to model “falsification,” rather than validation, based on the contention that models can only be invalidated (falsified). One common feature of most of the different interpretations of validation is that validation must involve new data not used in the construction of the model, i.e., “calibration is not validation” (Roache, 2009).

The DoD definition arguably presents a contradiction regarding validation of CEP models—especially *cellular* models—because they are typically developed as **general-purpose models**, i.e., without a specific COU in mind. When novel cell models are published, predictions of model outputs and derived quantities—for example, action potential shape, action potential duration (APD) restitution, ionic concentration transients and others—are usually compared to experimental data. This arguably does not constitute validation according to the DoD definition since no COU (intended use) has been prescribed. Incidentally, this could be considered a limitation of the DoD definition; see (Roache, 2009) for a discussion. Note though that regardless of the definition of validation, it is vital to recognize the importance of the COU in evaluation of a computational model. In particular, the COU must be specified for the “final” evaluation of a model, and any claim that a general-purpose model is a “validated model” cannot be justified, since it is the COU that determines what level of agreement between model and experiment can be deemed acceptable (Roache, 2009; National Research Council, 2012).

To distinguish between different types of evidence, we introduce the terminology **general validation evidence** to describe scientific evidence obtained by comparing model predictions with real world data *when no particular COU has been specified*. This could also have been called “baseline validation.” Examples of general validation for CEP models include initial validation of a novel general-purpose cell model (discussed in detail in section Cell Models), general testing vs. experimental/clinical data of previously published cell models (regularly carried out in the cardiac modeling community), and the comparison against data of activation patterns predicted by general-purpose ventricular, atrial or whole-heart models (discussed in section Organ-Level Models). There is almost unlimited scope for such evaluation, since modern CEP cell models are very complex, and therefore there is an ever-growing volume of literature incorporating general validation of CEP models.

We define **COU-driven validation evidence** using the DoD definition, as scientific evidence obtained by comparing model predictions with real world data for the purposes of evaluating the predictive capability of the model for a specific, prescribed, application (COU) of the model. A simple example of this would be comparing APDs of a model to experimental values, when the COU is prediction of drug effects on APD. Another example is comparison of whole-heart model predictions of number of phase singularities during ventricular fibrillation, against clinical data, when the COU is to use the model to understand mechanisms behind ventricular fibrillation (see section Organ-Level Models). We include in this category validation of model-derived quantities, including: drug pro-arrhythmic risk indices (see sections Cell Models and Organ-Level Models); sudden cardiac death (SCD) risk indices (see section Organ-Level Models); and ablation targets (see section Organ-Level Models).

Note that a terminology complication can arise when considering validation of patient-specific models, which are often generated using a workflow that may be mostly or fully automated. One could distinguish between validation of the simulation software *only*, and validation of model predictions using the full *workflow*; there is therefore a potential for different interpretations of what constitutes “model validation” in this context. In this paper, we will include validation of the full workflow (for example, evaluation of the predictive ability of a workflow that takes in patient imaging data and outputs a clinical prediction) within our broad interpretation of model validation.

### Comparator, quantities of interest, and method of comparison

Validation involves comparison of model predictions with real world data of some form. (Note that comparison against results of a *different* computational model is generally not considered validation, but see Roache, 2009 for a discussion). The **comparator** is defined as the source of the real world data. For CEP models this is usually experimental or clinical data. Important aspects of experimental comparators in CEP model validation include species, experimental conditions including temperature, and whether the data is historical (taken from the literature) or obtained from new experiments performed for the purpose of model validation. Important aspects of clinical comparators include patient demographics, patient cardiac myopathies, and co-morbidities. For patient specific CEP models that make patient specific clinical predictions, the validation comparator has to be clinical data taken from the same patient (distinct to the data used for personalisation of the model). Regardless of whether the comparator is experimental or clinical data, there are often significant challenges to obtaining high quality data, especially *in vivo* data under physiological conditions, which can impose severe constraints on the ability to perform high quality validation. These experimental/clinical challenges are covered elsewhere in the CEP literature, and therefore will not be a main focus of the present review.

Another important aspect of validation is which outputs of the model, or derived quantities—here referred to as **quantities of interest (QOIs)**—are compared to the real-world data. Commonly validated QOIs for cell models include transmembrane voltage and the APD restitution curve. For whole-heart models, validation QOIs can be global (e.g., the ECG) or local (e.g., activation patterns). Validation using global QOIs only provides indirect evidence on the credibility of local QOIs.

There are various possibilities for the **method of comparison** between the model and comparator. (Oberkampf et al., 2004) provide a good introduction to this topic; here we only provide a very brief overview. The comparison can be qualitative (often the case in physiological modeling) or quantitative. If quantitative, the comparison could take into account experimental error, model uncertainty, both, or neither. Model uncertainty is accounted for by performing uncertainty quantification (UQ), where uncertainty in model parameters (due to, for example, measurement uncertainty or inherent physiological variability) is quantified using probability distributions, and then the resultant uncertainty in the QOI(s) are computed (Smith, 2013; Mirams et al., 2016). Various validation metrics for quantifying the difference between experimental data and model predictions taking into account error estimates and simulation uncertainty have been proposed in the engineering literature; (see e.g., Oberkampf and Barone, 2006). For some CEP model-derived outputs such as risk indices or model-based biomarkers, other analytic or statistical comparison methods (different to those used in traditional model validation) may be appropriate, such as measures of specificity and specificity, receiver operating characteristic (ROC) curves, biomarker validation methods, etc.

Sometimes a CEP model is stated as matching known physiological phenomena, for example in statements such as “the AP shows the characteristic spike notch dome architecture found for epicardial cells” (ten Tusscher et al., 2004) or discussion of re-entrant waves breaking up into sustained fibrillation under pro-arrhythmic conditions (Krishnamoorthi et al., 2014). This is perhaps not validation *per se*, as there is no explicit comparator—or more precisely, arguably not validation according to the engineering/DoD understanding of validation; it is arguably “epistemic validation” using the broader definition of Patterson and Whelan (2017). Nevertheless, it is important and relevant evidence for assessing the model's predictive ability for a COU. This type of evidence, which we will refer to as **reproduced phenomena**, may be especially important in evaluation of biological models since biological systems exhibit emergent phenomena, and therefore a powerful test of a model is whether such it predicts such phenomena.

### Validation of multiscale models

For multiscale models we can distinguish between evidence at different spatial scales, and in particular at which scales validation was performed (see Figure 2). For a multiscale model of the whole-heart, there may be validation evidence available for model sub-components (i.e., all sub-cellular models and the cell model), and/or for the system-as-a-whole (whole-heart model). If validation is only performed for sub-models but *not* the overall system, credibility of system-level predictions is founded (perhaps implicitly) on the sub-model validation results *and* belief in the theory underlying how sub-models interact. For example, most cardiac cell models assume that ionic currents are independent and can therefore simply be added together. System-level validation may be especially important with physiological models, since physiological systems exhibit emergent behavior that cannot be predicted from understanding all sub-system behavior. “Hierarchical validation,” in which validation is performed for all model sub-components, sub-systems *and* the entire system, is recommended in the engineering validation literature so that the model provides the “right answer for the right reasons” (Hills et al., 2008).

Often, validation is performed at one scale to provide confidence that the model is sufficiently credible for it to be used as a sub-model in a larger scale (e.g., develop a cell model, perform validation of cell model, and then proceed to tissue model if validation results are favorable). Even if this is the case, the sub-model validation results may be relevant in evaluation of the final model for a COU.

It should now be clear how a CEP model may be supported by **multiple sources of credibility evidence, taken from model evaluation at multiple scales** (see Figure 2). Table 1 lists different sources of evidence and provides examples for ion channel, cell and organ-level models. We reiterate that we are not making any assertions regarding what evidence is *necessary* when assessing cardiac models for a COU. Our motivation is simply to describe how multiple sources of evidence may exist and be relevant when assessing the credibility of a CEP model for a specific COU. Confidence in a model tends to increase with the body of evidence available to support it (Patterson and Whelan, 2017). Therefore, when a complex model is evaluated, ideally the model should be treated as a “glass box” (the opposite of a “black box”), so that the most informed decision is made. Any or all of the types of evidence in Table 1 may be relevant in glass box cardiac model evaluation. The most important source of evidence for a whole-heart model will likely be organ-level COU-driven validation evidence, if available. Strong validation results of the full model, if highly “applicable” (Pathmanathan et al., 2017) to the COU, reduce the relative importance of the other factors (including reducing the need for evidence supporting model assumptions (Patterson and Whelan, 2017).

**Table 1:** Different types of evidence relevant to the credibility of a cardiac EP model, with ion channel, cell, and organ-level examples.

**Category**	**Type of credibility evidence**	**Examples**		
		**Ion channel**	**Cell model**	**Organ-level model**
*Category 1*	Evidence regarding validity of model assumptions or supporting the model formulation	Successes of Hodgkin-Huxley formulation for modeling ion channels—see section Ion channel models	Evidence supporting the formulation of cell membrane as a parallel resistor-capacitor electric circuit	The successes of the bidomain equations, in particular predictions made that were later experimentally observed—see section Organ-level models
	Evidence regarding accuracy/fidelity of model parameters/inputs	Evidence supporting accuracy of steady-state inactivation parameters—see section Ion Channel Models	Rationale behind standard choice of membrane capacitance equal to 1 uF/cm^2^.	Evidence on fidelity of geometry used and on fidelity of fiber/sheet specification—discussed in section Organ-Level Models.
*Category 2*	Calibration results	Results showing agreement between ion channel model and experimentally recorded current-voltage relationship when ion channel parameters are calibrated using this data	Results showing agreement between the model action potential and experimental recordings when maximal conductances are tuned to achieve the match	Results showing activation patterns match experiment if fast sodium current maximal conductance (which controls conduction velocity) chosen to maximize agreement
*Category 3*	Reproduced (emergent) phenomena	Simulation results demonstrating that a rapid sodium current model can exhibit damped oscillations	Simulation results demonstrating that a cell model reproduces action potential spike and dome morphology	Simulation results demonstrating that ECG predicted by a heart and torso model exhibits realistic-looking QRS complex and T wave
	General validation results	Comparison of a general-purpose ion channel model predictions to new voltage-clamp data not used in the construction of the model.	Comparisons of model results with experimental data for a novel general-purpose cell model, e.g., all such results in O'Hara et al. (2011). Discussed in detail in section Cell Models	Excitation patterns of general purpose bi-ventricular model compared to experimental/clinical data. ECG of general-purpose heart and torso model compared to experimental/clinical data.
	COU-driven validation results	Evaluation of a hERG model to predict pharmaceutical pro-arrhythmic risk	Evaluation of a cell model-based biomarker to predict pharmaceutical pro-arrhythmic risk (e.g., CiPA, discussed in section Cell Models)	Number of phase singularities during ventricular fibrillation (VF) compared to clinical data, when the model will be used to understand mechanisms behind VF—see section Organ-Level Models. Clinical evaluation of a whole-heart model which uses patient-specific information to predict optimal ablation targets to terminate arrhythmias—see section Organ-Level Models

## Credibility of CEP models at different spatial scales

We now discuss credibility evidence of CEP models at each of the spatial scales. The scope of the following review is limited to the most common types of CEP model: zero-dimensional models (i.e., systems of ODEs) of ionic channels and of the cell, and tissue/organ models that utilize the monodomain or bidomain formulation. Therefore, models that explicitly represent the spatial structure of ion channels or cardiac myocytes are out of scope of the review, including molecular dynamics models. Due to space limitations, we will only discuss ion channel models; other types of sub-cellular model such as calcium handling models are not included. We only consider models which are at least partially motivated by bio-physical understanding, excluding phenomenological models, or statistical models such as those developed using neural networks or machine learning techniques. We re-iterate that this paper is focused on electrophysiology only; models of cardiac mechanics or hemodynamics are out of scope, although similar principles are expected to apply. Note that the scope of the following review is still quite broad and it is therefore not possible to describe or cite all publications that have performed validation of CEP models. The papers cited below were chosen to provide selected examples of approaches to CEP model validation.

### Ion channel models

There is a long history of modeling the dynamics of transmembrane ion channels using the Hodgkin-Huxley (HH) formulation (Hodgkin and Huxley, 1952). In the HH formulation, transmembrane current is taken to be the product of a maximum conductance, dynamic gating variables representing probabilities of channels gates being in an open state, and a driving force. Gating variable dynamics are modeled using ODEs, with dynamics determined by the voltage-dependent steady-state activation/inactivation and voltage-dependent “time constant” relationships for each gating variable (see Figure 1, which includes the equations for a HH formulation of the rapid sodium current with three gating variables *m, h*, and *j*). The HH formulation has in fact become so integral to cardiac electrophysiology that experimentalists regularly present data by publishing HH-based model parameters. Markov models of ionic currents are a more general formulation. For more details (see e.g., Fink et al., 2011).

**Figure 3:**
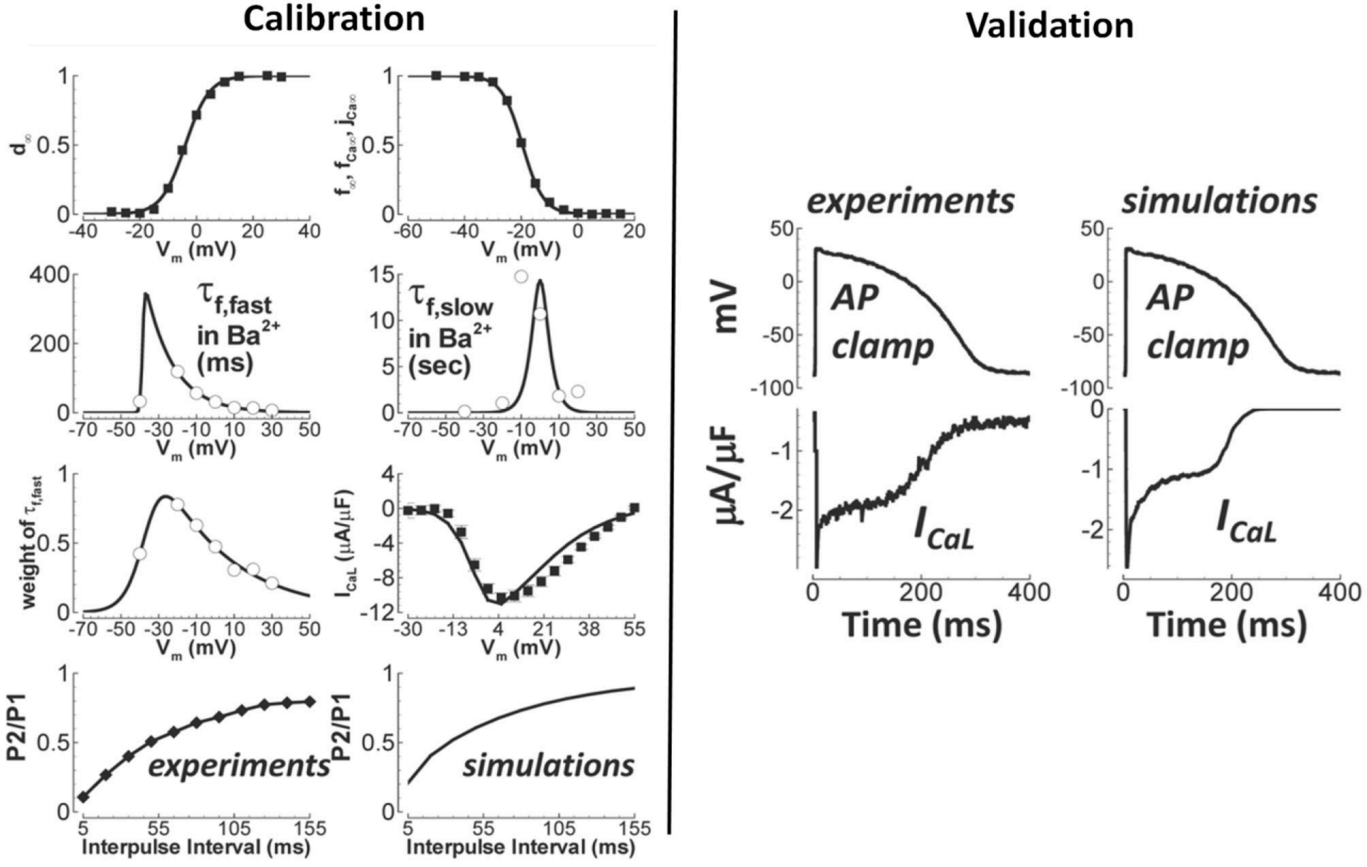
Calibration and validation of the L-type Ca^2+^ current of O'Hara et al. (2011). Left figures show calibration results (circles/squares/diamonds—experiment; solid lines—simulation), including fitting of steady-state activation and inactivation (top row) and time constants (second row). Right figures are qualitative validation of the formulated I_CaL_ model by comparison of simulation and experiment under an identical action potential clamp. Quantitative validation of peak current is also provided in original paper. (Adapted from Figure 1 of O'Hara et al. (2011) with permission under Creative Commons license).

Generally speaking, validation of novel ion channel models is *not* common practice (Fink et al., 2011). Here, we are referring to validation of the novel channel in isolation, not as part of a larger cell model. While voltage clamp data is used to develop and calibrate the models, those calibrated models are typically not then tested to new data. In fact, generally simulations are not even performed to show that the models predict the voltage-clamp results that they were based on, and surprisingly, simulations of voltage clamp protocols from which parameters are derived do not necessarily match the original data (Carro et al., 2017). (This can happen for a variety of reasons, such as the assumption of inactivation being much faster than activation not holding). Such observations demonstrate the value of ion channel model evaluation including validation. It can be difficult to determine in publications if results presented correspond to validation, because calibration and validation are often not clearly separated in presentation of results. An example of genuine validation is (Yang et al., 2015), in which validation of a new model of the late sodium current *I*_*NaL*_ is performed by comparing model predictions of the *I*_*NaL*_ current-voltage relationship under a slow depolarising voltage ramp, against experimental recordings under the same protocol. Another is the L-type calcium current model in O'Hara et al. (2011). As shown in Figure 3, validation of the calibrated *I*_*CaL*_ model was performed by comparing model with experimental data using an action potential clamp protocol. (Beattie et al., 2017) proposes a novel approach to developing cell-specific models of the rapid delayed rectifier potassium current *I*_*Kr*_. Eight seconds of data using a novel sinusoidal voltage clamp protocol was used to calibrate the cell-specific *I*_*Kr*_ models, which were then validated against 5 min of data taken from the same cell, covering a range of voltage clamp protocols.

When no validation evidence is presented, the credibility of novel ion channel models is essentially founded—often implicitly—on a range of other factors, including the maturity of the HH formulation and related historical evidence, calibration evidence, and evidence regarding the accuracy of identified parameters. We discuss these in the remainder of this section.

The model of squid giant axon excitability proposed by Hodgkin and Huxley (Hodgkin and Huxley, 1952) is considered one of the greatest successes in twentieth century biophysics (Häusser, 2000; Schwiening, 2012). This is due to the ability of the relatively simple set of equations to reproduce a variety of phenomena (Häusser, 2000) and the fact that the HH modeling approach was then successfully applied to a wide range of excitable cells, including cardiac cells (Noble, 1962). The ideas and equations behind the HH model are now standard building blocks in electrophysiology (Schwiening, 2012). HH-based cardiac models have contributed greatly to understanding of cardiac electrophysiology, with various predictions made using cardiac models that were later experimentally verified. Examples include the existence of non-sodium inward currents and stoichiometry of the Na^+^/Ca^2+^ exchanger; see (Noble, 2011) for a detailed review. However, despite these successes, there are several caveats that should be stated regarding use of a general HH formulation for a given ion channel. First, for some ion channels and some applications, such as the rapid delayed rectifier potassium current *I*_*Kr*_ and drug-binding applications, a Markov model based approach may be more appropriate (Clancy and Rudy, 1999). Additionally, there is still a lack of consensus and ongoing research into a variety of details of specific formulations. For example, for the fast sodium current *I*_*Na*_, while the originally-proposed (Hodgkin and Huxley, 1952) and commonly-used *m*^3^ formulation of activation can be argued to have a justification at the molecular level (Armstrong, 2006), it is unclear how to simultaneously represent the various modes of *I*_*Na*_ inactivation (fast, slow, and persistent; Nesterenko et al., 2011). Similarly, for the L-type calcium channel *I*_*CaL*._, there is not a unique approach to simultaneously quantifying both voltage- and calcium-dependent inactivation (Grandi et al., 2010).

Once a HH-based model formulation is proposed and justified, model parameters need to be estimated. This includes parameters representing the voltage-dependent steady-state activation/inactivation and time-constant functions for each gate, which are estimated using voltage clamp data. Evidence on the accuracy of these parameters is important in evaluating model credibility, especially if no validation is performed. However, before asking about the accuracy of parameter values, one can ask if ion channel model parameters are uniquely identifiable from experimental data in the first place. A parameter cannot be claimed to be accurate if it is provably unidentifiable given the data. Although the methodology for nonlinear model identifiability has been extensively studied (Rothenberg, 1971; Jacquez and Greif, 1985; Walter and Pronzato, 1996), their utilization in the field of CEP modeling has been limited. The conditions under which model parameters can be identified has been studied in the context of single current sub-models (Beaumont et al., 1993; Wang and Beaumont, 2004; Lee et al., 2006; Csercsik et al., 2012; Raba et al., 2013) and more recently incorporated into a multi-scale framework using a simplified action potential model (Shotwell and Gray, 2016).

Returning to parameter estimation, voltage-dependant *steady-state* (in)activation relationships for many currents are typically well-approximated using sigmoidal functions using standard voltage clamp protocols, however obtaining data for accurate characterisation of voltage-dependent time constants is considerably more difficult. Assumptions underlying voltage clamp protocols should be well understood by model developers and may be questionable for protocols used to identify certain parameters (e.g., the assumption that inactivation is much faster than activation for protocols used to identify *I*_*Na*_ steady state gating activation parameters Csercsik et al., 2012). Manual recording from single cells using well-established voltage clamp protocols remains the gold standard for obtaining high-quality current-voltage relationships (Elkins et al., 2013). Nevertheless, there are numerous (often “hidden”) details regarding the specific procedures and protocols in the laboratory to isolate individual currents and to minimize recording artifacts (e.g., accounting for liquid junction potentials and capacitive compensation). Technical advances has improved the ability to measure rapid transients, however, it is still not possible to characterize activation for the fast sodium current steady-state activation from adult myocytes under physiological conditions (Berecki et al., 2010). Experimental reproducibility and variability between cells also present challenges (Pathmanathan et al., 2015). Regarding the voltage dependence of time constants, there is not even consensus on the appropriate functional forms, unlike steady state parameters. In general, fits to time constant data are much poorer than to steady-state (in)activation data [compare steady state and time constant fits in (e.g., ten Tusscher et al., 2004) or (O'Hara et al., 2011); also see Figure 3]. The quality of such fits is rarely quantified.

Overall, if a novel ion channel model is developed but no validation is performed, given the numerous issues described above there may be significant uncertainty regarding the true predictive capability of the ion channel model. This is especially true for simulations using conditions that are quite different to the conditions used for model calibration. Often, however, novel ion channel models are developed as one component of a cell model, and validation is instead performed at that level, as described in the next section.

### Cell models

Regarding validation of cardiac cell models, we first make two remarks. First, it should be noted that the majority of cell models are developed as general-purpose tools, as opposed to for a specific COU. Any initial testing against experimental data of a general purpose cell model therefore falls under the category of “general validation.” Second, it can be especially difficult to determine in publications whether results presented are obtained by calibration or are genuinely validation evidence. For example, simulated and experimental action potentials may be presented in papers to demonstrate a close match between simulation and experiment, but it can be unclear if any parameters (e.g., ion channel maximal conductances) were tuned to obtain the match.

There is an enormous range in the extent of general validation performed when novel cell models are published. They can vary in terms of which model outputs are compared to experiment, which pacing protocols are applied, the source of the experimental/clinical data, and the type of comparison between model and experiment (e.g., qualitative vs. quantitative). As an example, for validation of their human cell model, (ten Tusscher et al., 2004) first present action potential and calcium transient time courses under 1 Hz pacing, stating how the AP reproduces the characteristic spike notch dome of epicardial cells and the calcium transient reproduces the experimentally observed rounded-off triangular shape (“reproduced phenomena” evidence as discussed in section Why Trust a Computational Model?). They then semi-quantitatively compare several AP properties and diastolic/systolic calcium concentration with experiment, qualitatively compare APD restitution and conduction velocity restitution results with experiment, as well as present several other validation-related results, including at the tissue level (after coupling the cell model with the monodomain equations). This is arguably more extensive validation than presented for *most* other cell models. The most comprehensive set of validation tests for a new cell model is, as far as we aware, that presented in the original O'Hara-Rudy-dynamic (ORd) model paper O'Hara et al. (2011), in which validation was performed for all of the following QOIs: AP shape under multiple pacing rates, resting voltage, maximum voltage, maximum upstroke velocity, APD restitution properties (steady state; dynamic; with and without channel-specific blockers; single cell and in tissue), APD alternans and accommodation, AP shape with induced early after-depolarisation (EAD), peak intracellular sodium and calcium ion concentrations at multiple rates, calcium transient at multiple rates, and various current voltage relationships under various voltage/potassium/sodium/calcium clamps. This extensive validation, together with the use of human data for model development, are reasons why the ORd model is one of the most highly regarded of modern cell models, although we emphasize that even this model should not be considered a “validated cell model,” both because of the issues with such terminology (see section Validation and Roache, 2009), and also because of certain ways it does not match clinical observations (Mann et al., 2016; Dutta et al., 2017).

In general it is important to note that modern cell models may simulate dozens of quantities (i.e., have dozens of state variables), of which usually only a handful have been directly compared to experimental data; this is certainly true even of the ORd model. Credibility in QOIs not compared to experiment is therefore based on “indirect” validation. It should also be appreciated that most cell models are typically *not* validated using data directly related to the initiation and maintenance of arrhythmias, although there are notable exceptions such as the validation involving EADs in O'Hara et al. (2011) or Nordin and Ming (1995) and involving reentrant waves in ten Tusscher et al. (2004).

The above are all examples of general validation; next we consider validation of cell models for a prescribed COU, i.e., COU-driven validation. For single cell cardiac models, the application (i.e., COU) with the greatest current research interest is prediction of proarrhythmic risk of novel pharmaceutical compounds (Davies et al., 2016). (Davies et al., 2012) develop an ensemble of 19 cell models calibrated to data from 19 dogs for this COU. For validation, they first compare model predictions of drug effect on action potential shape against experimental data (using various compounds). They then test the ability of the model ensemble to predict—blinded—whether a drug will cause AP shortening, prolongation, or have no effect, on a test set of 53 compounds and using measures of sensitivity, specificity and predictivity. Other CEP model-based biomarkers have also been recently proposed (Mirams et al., 2011; Passini et al., 2017), and have been evaluated against risk classifications scores using test sets of compounds. This application area has matured rapidly, and recently regulators, academia and the pharmaceutical industry have come together in the Cardiac *in vitro* Proarrhythmia Assay (CiPA) program (Cavero and Holzgrefe, 2014; Colatsky et al., 2016). The aim of the CiPA program is to develop a novel framework for assessing proarrhythmic risk. The proposed framework involves a series of predominantly nonclinical assays, one of which utilizes a cardiac cell model to integrate drug ion channel effects to the action potential level. The ORd model is being modified for this purpose, and the ultimate aim is to develop a model-based metric that converts drug ion channel effects into a predictive risk index (Dutta et al., 2017). Twelve drugs with well-characterized risk are being used for model and metric development, and the final metric will be validated (in a blinded fashion) using 16 different drugs with well-characterized proarrhythmic risk.

When no validation evidence is available for a cell model, which may be the case for a novel—or considerably modified—cell model, credibility of model predictions is essentially founded, perhaps implicitly, upon multiple factors. This includes the consensus view that the cell membrane can be modeled as a parallel resistor-capacitor electric circuit (Cole and Moore, 1960; Mauro et al., 1970), together with any evidence supporting credibility of each of the sub-cellular models incorporated (i.e., as discussed in section Ion Channel Models), and any calibration evidence (e.g., ability to reproduce AP shape or characteristics when model parameters are selected accordingly). In this case a lot of subject matter expertise may be required to interpret and to judge reliability of predictions.

### Organ-level models

Tissue- and organ-level simulations have been used for many years and with great success in basic science applications (Trayanova et al., 2006; Bishop et al., 2009). These models involve the solution of the bidomain or monodomain equations (Tung, 1978; Henriquez, 1993; Neu and Krassowska, 1993; Bourgault et al., 2009), incorporating one or more specific cell models, on a computational mesh that approximates the geometry of interest (which can be a 2D monolayer (ten Tusscher et al., 2004), 3D slab of tissue, the atria (Seemann et al., 2006; Zhao et al., 2013), the ventricles (Plank et al., 2009) or the whole heart (Deng et al., 2012; Baillargeon et al., 2014). It is also possible to model the heart in a conductive medium, such as saline bath or the torso, which allows the electrocardiogram and defibrillation to be simulated (Aguel et al., 1999; Richards et al., 2013; Zemzemi et al., 2013; Okada et al., 2015). Tissue-level parameters that need to be prescribed include intra- and extra-cellular conductivities (dependent on the local fiber and sheet directions Legrice et al., 1995). For more details (see, e.g., Lopez-Perez et al., 2015), which reviews 60 3D cardiac models developed over the past fifty years.

In fact, the methodology for tissue- and organ-level simulation studies is so well-established that simulation studies are routinely published in which a model is used for EP research but no validation results are presented, and no other rationale for credibility is explicitly presented. For such studies, the credibility of the model predictions is essentially based—implicitly—on the following: (i) confidence in the model governing equations (including historical evidence supporting the bidomain formulation); (ii) confidence in the cell model used; and (iii) the accuracy/fidelity of model parameters and geometrical inputs. We discuss each of these below.

First, we note that bidomain equations have a strong biophysical basis, being mathematically derived through a formal homogenisation of an underlying set of governing equations derived from Maxwell's equations (Neu and Krassowska, 1993). The underlying anatomical and physiological assumptions are mostly considered reasonable, although there remains ongoing research into alternative formulations that may better represent electrical propagation through myocardium, for example the fractional diffusion model of Bueno-Orovio et al. (2014), the alternative homogenisation derived by Richardson and Chapman (2011), or the hyperbolic bidomain model of Rossi and Griffith (2017). The bidomain equations reduce to the monodomain equations under the assumption that the intracellular and extracellular conductivity tensors are aligned. While this is known to not be the case in cardiac tissue, in the absence of extracellular stimuli (such as defibrillatory shocks) solutions of the monodomain equations can be very similar to those of the bidomain (Potse et al., 2006; Clayton et al., 2011). Perhaps the strongest evidence supporting the use of the bidomain equations are the numerous historical examples of quantitative predictions from bidomain simulations that have been reproduced experimentally, including complex phenomena that were predicted by simulation studies and only later observed experimentally. The most famous example regards specific virtual electrode patterns: simulations preceded experiment in predicting that unipolar excitation can result in a “dog-bone” shaped virtual cathode with regions of hyperpolarisation (virtual anode) in the vicinity of the virtual cathode (Sepulveda et al., 1989; Wikswo et al., 1991, 1995). This unexpected phenomenon is the result of the unequal anisotropy ratios of the intracellular and extracellular conductivity tensors. (Wikswo and Roth, 2009) provide a detailed review and numerous other examples of bidomain simulations matching experiment.

Credibility of tissue-level predictions is also dependent on the specific cell model used in the simulations. Credibility of cell models was discussed in section Cell Models. However, note that validation at the cell level does not necessarily imply that simulations will reproduce tissue-level phenomena. For example, (Gray et al., 2013) measured the action potential upstroke shape during propagation and found that it differed from that predicted in tissue simulations using a variety of cell models. (Uzelac et al., 2017) show that current cell models when incorporated into tissue level models do not reproduce the voltage and calcium dynamics of alternans. In addition, it is fairly common to adjust cell model parameters in tissue simulations (e.g., to shorten APD when simulating fibrillation Bishop and Plank, 2012), without any “re-validation” of the modified cell model; for such cases, it is unclear how much the previous cell model validation results can be relied upon. It is also increasingly common to re-calibrate cell model parameters in an organ-level model using data taken from intact tissue, including clinical data (e.g., Keldermann et al., 2008); again, it is unclear the extent that the body of previous validation results holds. We will return to this subject in the discussion.

The third factor especially relevant to model credibility when no validation results are available is the accuracy/fidelity of model parameters and other inputs. In regards to parameters we refer to Clayton et al. (2011), which provides a review of the challenges of estimating parameters in the bidomain equations. Note though that when estimating personalized parameters from clinical data for patient specific models, questions can be raised on the identifiability and accuracy those parameters; see (Chabiniok et al., 2016) for a general discussion. Here we focus on geometrical inputs. In organ-level simulations, an important factor that may require consideration when evaluating credibility is the anatomical fidelity of the computational mesh. There are a range of possibilities, from use of simple truncated ellipsoids (Vetter and McCulloch, 1998) to image-based meshes. Meshes vary in terms of the anatomical detail included. For example, they may include ventricular endocardial structures such as papillary muscles and trabeculae (Bishop et al., 2010); atrial endocardial structures such as fossa ovalis (Seemann et al., 2006); myocardial blood vessels (Bishop et al., 2010); and/or the Purkinje system (Romero et al., 2010; Bordas et al., 2011). The appropriate level of detail for specific applications is not yet clear; in particular there is ongoing research into the role of microstructure on the initiation, maintenance and termination of fibrillation (Bishop and Plank, 2012; Connolly et al., 2017). As well as geometry, there is a question on the fidelity of the prescribed fiber and sheet orientations. This can be estimated using DT-MRI imaging data (Mekkaoui et al., 2012); however DT-MRI data can be noisy due to partial volume effects and sensitive to motion artifacts (Bishop et al., 2009; Dierckx et al., 2009). An alternative approach is to use a “rule-based” method (see **Figure 5**, later, for an example), in which a mathematical algorithm is used to generate fiber and sheet architectures [see e.g., (Potse et al., 2006; Bishop et al., 2010; Bayer et al., 2012) for ventricles or (Krueger et al., 2011; McDowell et al., 2012) for atria], and has been shown to provide results that are very similar to those based on DT-MRI (Bishop et al., 2009; Bayer et al., 2012), but may not correctly capture fine-scale details such as fiber direction near the apex, around vessels or near infarcts. Therefore, either way, there may be considerable uncertainty about the true fidelity of the prescribed fiber/sheet directions, which may impact credibility of predictions of quantities expected to be sensitive to anisotropy.

Next, we move on to validation of organ-level models. The ability to perform validation of such models is of course heavily constrained by difficulties in obtaining the necessary experimental or clinical data for model validation, and therefore the vast majority of validation of organ-level models has been limited to heart surface potentials. Heart surface potential data can be obtained from a variety of measurement modalities, including transmembrane voltage recorded from glass microelectrodes or using fluorescent dyes (e.g., optical mapping), or extracellular electrograms using electrode plaques, socks, baskets, or other mapping systems (contact and non-contact). These measurements vary in their spatial resolution from a single site to hundreds or thousands of sites. Each modality has its advantages and disadvantages; for example optical mapping provides very high spatial resolution but low voltage fidelity, and is always *ex vivo* for human tissue and only *in vivo* with great difficulty for animal experiments (Dillon et al., 1998). In contrast, extracellular electrograms can be used to obtain *in vivo* data but at lower spatial resolution. With the exception of the transmembrane microelectrodes, all modalities do not directly measure transmembrane voltage, which can lead to difficulties in achieving a like-for-like comparison between simulation and experiment. This can be remedied in the computational model. For example, fluorescent signals from optical mapping are different than transmembrane signals in that they have a longer upstroke (Gray, 1999), which was determined to be a result of photon scattering (Hyatt et al., 2003), which led to the development of CEP models that also simulated fluorescence with scattering to enable like-for-like comparisons (Bishop et al., 2007; Roth and Pertsov, 2009).

**Figure 4:**
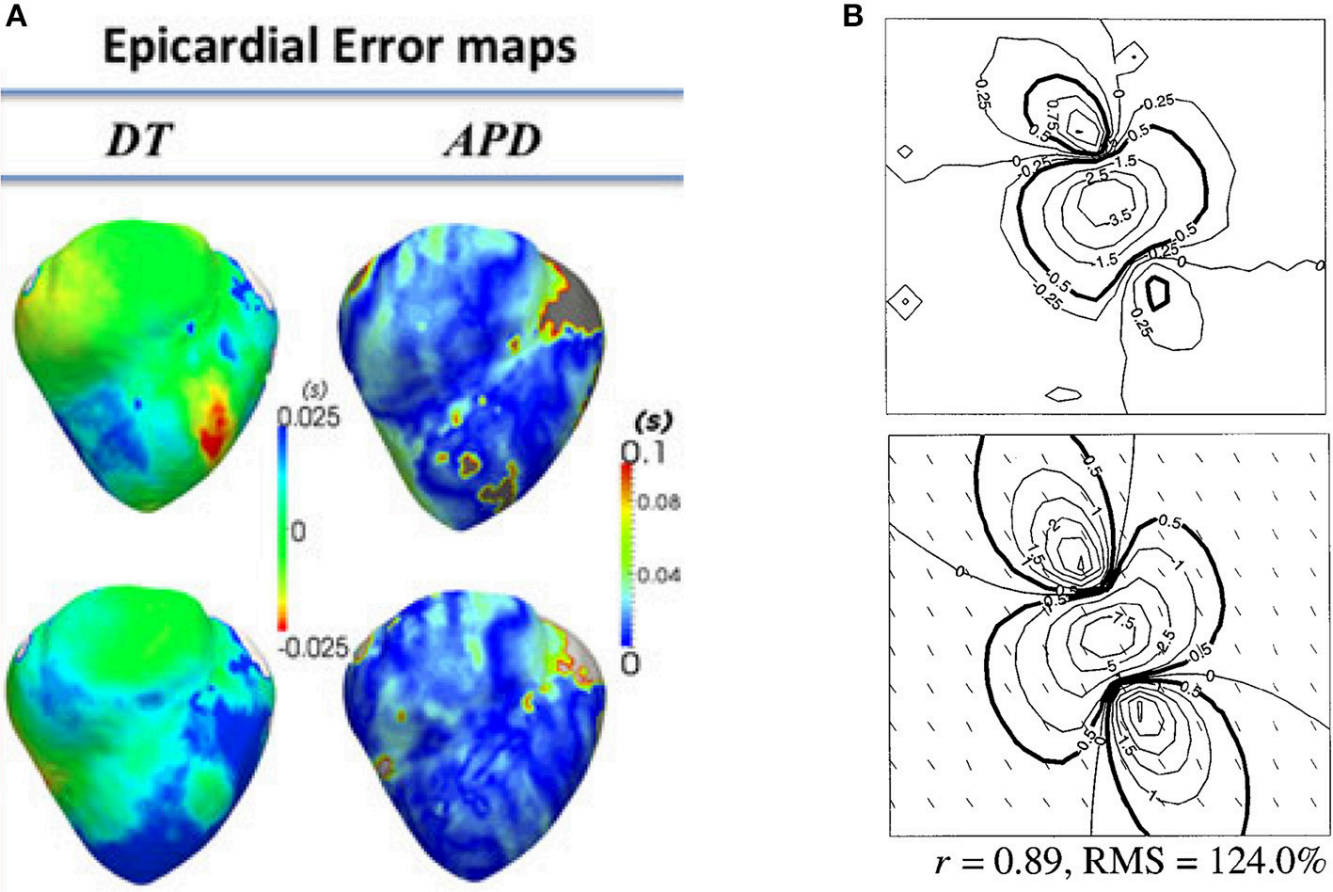
Examples of quantitative validation of organ-level models. **(A)** Error maps (i.e., difference between model and experiment; here optical mapping-derived experimental data) for depolarisation time (DT) and APD (top row—pacing on left ventricle epicardium; bottom row—pacing on right ventricular endocardium) (Reproduced with permission from Relan et al., 2011). **(B)** Experimentally measured extracellular potential in mV using electrode plaque (top) compared to predictions of extracellular potential from bidomain simulations (bottom), with difference quantified using Pearson's correlation coefficient (r) and root mean squared (RMS) error. (Reproduced with permission from Muzikant et al., 2002).

Many groups have performed validation of organ-level CEP models using data obtained from these modalities. Here we will provide a few representative examples, to give a flavor of the possibilities for validation of surface potentials or derived quantities. (Relan et al., 2011) describe a framework for the functional personalisation of a porcine biventricular model using *ex vivo* porcine optical mapping data. As shown in Figure 4A, following calibration using optical recordings under one pacing protocol, they quantitatively validated predictions of epicardial APD and activation time, using optical recordings from the same heart under various different pacing scenarios. (Rodriguez et al., 2005) investigated the role of structural differences between right and left ventricles in vulnerability to electric shocks in the rabbit heart. The study used a combination of biventicular bidomain simulations and optical recordings from an experimental Langendorff-perfused rabbit heart. The setup enabled various QOIs to be qualitatively compared between simulation and experiment (to support the credibility of simulation-based results of the study), including post-shock transmembrane potential distributions on the epicardial surface, and the probability of tachyarrhythmia induction as a function of shock strength and coupling interval. (Muzikant and Henriquez, 1998) and (Muzikant et al., 2002) compare bidomain predictions with experimental results from the paced *in vivo* canine heart measured using a 528 channel electrode plaque. This study is notable because of the quantitative approach to the validation of spatial patterns, analyzing the root mean squared (RMS) error and Pearson's correlation coefficient between simulation and experiment, for extracellular potential and conduction velocity; see Figure 4B. (Niederer et al., 2010) use patient-specific biventricular electro*mechanical* models to investigate the relationship between the Frank-Starling mechanism and cardiac resynchronisation therapy (CRT) efficacy. To calibrate and validate the electrophysiological part of the electromechanical model, they use patient-specific clinical endocardial data obtaining using the EnSite™ cardiac mapping system. Clinical activation maps during sinus rhythm were used for model calibration, and activation maps under left ventricular pacing were used for validation of the calibrated model. (ten Tusscher et al., 2009) is a combined modeling and clinical study on the organization of ventricular fibrillation (VF) in the human heart. To support the credibility of the model used, epicardial excitation patterns are compared between model and clinical recordings obtained using a sock electrode, as is the time series of electrical activity at a fixed location. Dominant frequency of the time series is used for quantitative comparison between model and experiment. In addition, numbers of wavefronts and number of phase singularities over time are also compared. These quantities are convenient for condensing the complex spatio-temporal behavior of VF into simple time-series, useful for potential quantitative validation of very complex behavior.

**Figure 5:**
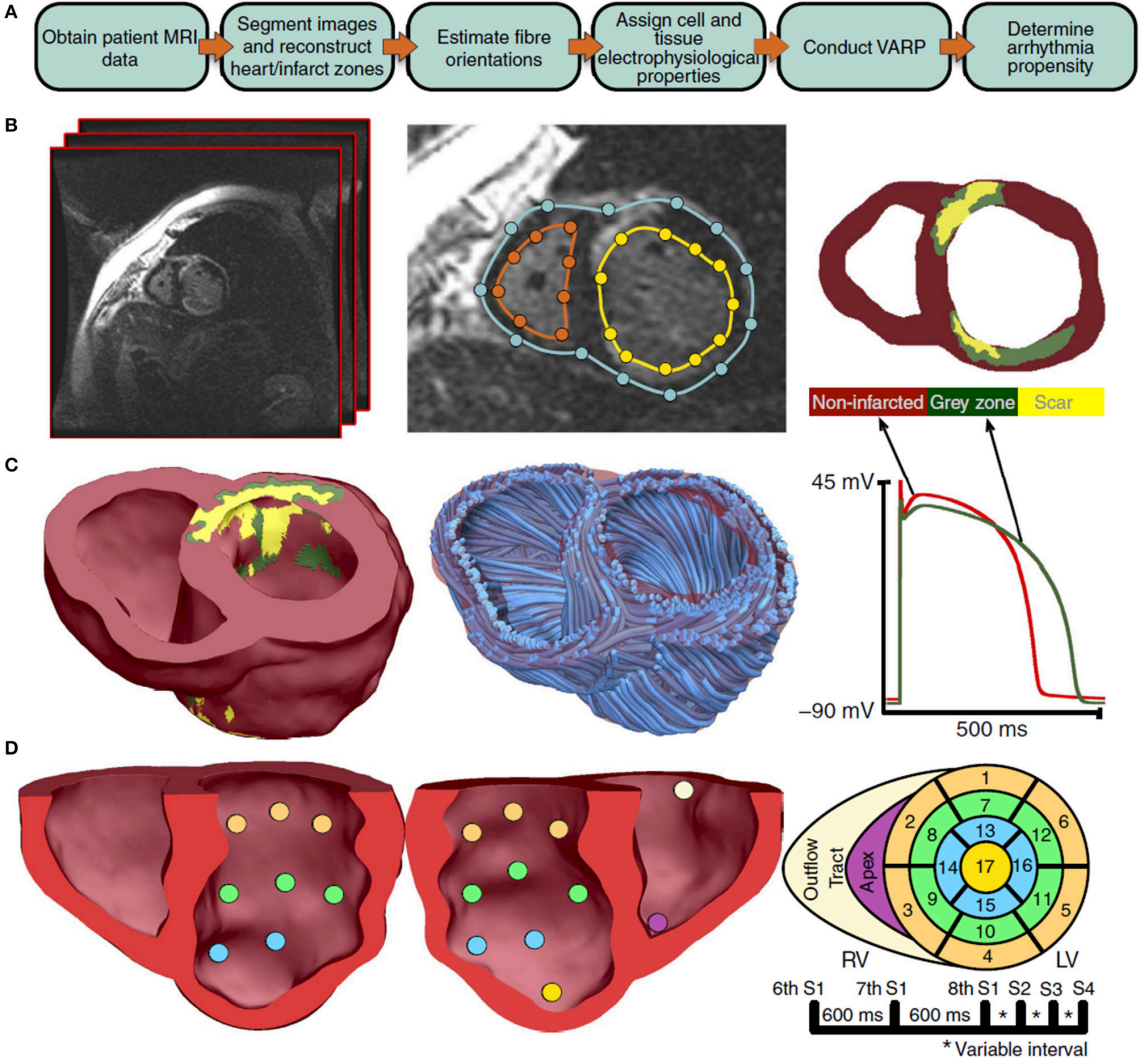
Overview of process used to predict sudden cardiac death risk in Arevalo et al. (2016). A flowchart of the pipeline is shown in sub-figure **(A)**. MR images are segmented [sub-figure **(B)**] to develop a patient-specific computational model which includes regions of scar tissue and border zone (“gray zone”) [sub-figure **(C)**]. A rule-based approach is used to specify fiber directions. The model is paced from 19 sites [sub-figure **(D)**] and with various pacing protocols and assessed for whether ventricular tachycardia is inducible. (Reproduced from Arevalo et al., 2016 with permission under Creative Commons license).

Finally, we consider validation of organ-level CEP models with a specific clinical application; in particular, where a model is proposed to be used in clinical decision-making. One proposed application of CEP models is to use patient-specific simulations for risk stratification of patients with myocardial infarction, to determine which patients are at risk of SCD and therefore should receive prophylactic implantable cardioverter defibrillator (ICD) implant, as described in Arevalo et al. (2016). As illustrated in Figure 5, the software developed for this application uses patient-specific MR data to generate a biventricular mesh which includes regions of scar tissue and border zone. Electrical activity is simulated using the monodomain equations with the cell model of ten Tusscher et al. (2004). Various pacing protocols are virtually applied to determine if ventricular tachycardia (VT) is inducible, and if so the patient is classified as being at risk of SCD. We highlight two sets of validation results relevant to this model. The first, presented in Deng et al. (2015), is validation of epicardial excitation maps, for a swine version of the model, against swine data obtained using sock electrodes. The second, presented in Arevalo et al. (2016), describes a retrospective clinical study performed to test the risk index. In this study, the workflow described above was applied on a number of patients who had had ICD implant, and the risk classification as predicted by the model was compared to the clinical endpoint of ICD appropriately firing (or cardiac death). This is another form of (COU-driven) model validation, and of course it is a very strong form of validation because the QOI that is evaluated is the final QOI to be used in decision-making (i.e., risk index). Since it involves a clinical study, for this type of validation the appropriate quantitative analysis method is statistical; see (Arevalo et al., 2016) for details.

Other studies have proposed that related model-based tools could be used for prediction of ablation targets for patients affected by atrial fibrillation (AF) (McDowell et al., 2015), left atrial flutter (LAFL) (Zahid et al., 2016), or VT (Ashikaga et al., 2013). The proposed process for predicting ablation targets for all three arrhythmias is very similar to the process described above: an anatomically patient-specific model of the atria or ventricles is generated using MR data, and virtually interrogated to determine if AF/LAFL/VT is inducible. If so, ablation sites can be predicted that render AF/LAFL/VT non-inducible (a method for doing so is described in Zahid et al., 2016). (Zahid et al., 2016) presents a retrospective clinical study in which predicted LAFL ablation sites using the patient-specific atrial models are compared to the clinically ablated sites. (Chen et al., 2016) present a related workflow for developing patient-specific cardiac models, with personalisation of some functional parameters as well as anatomical personalisation. They perform validation by comparing model predictions of VT inducibility and re-entrant circuits to results from clinical VT stimulation studies.

Finally, similarly to cell models as discussed in section Cell Models, whole heart models have also been proposed to be used to predict drug-induced arrhythmogenic risk. (Okada et al., 2015) proposed that a heart and torso model which simulates the ECG can be used to integrate *in vitro* ion channel assays. The drug concentration at which Torsades de Pointes is induced in the simulated ECG is the proposed biomarker, and the predictive ability of this biomarker is tested using data for 12 drugs with well-characterized risk.

## Discussion

In this paper, we have categorized and discussed different types of evidence that could be used as a basis for the credibility of a CEP model. Our aim was to provide clarity on the body of evidence that typically is relevant (and often implied) in the evaluation of CEP models. As we transition into the era of Digital Health, there is a need for a systematic, rigorous and well-established methodology for justifying and assessing the credibility of computational models with biomedical applications. Current efforts toward these goals (ASME, 2017) are focused on “physics-based” models that have so far had most impact in medical devices applications (Pelton et al., 2008; Angelone et al., 2010; Stewart et al., 2012). However, these modeling fields are very different to physiological modeling in terms of model complexity, multi-scale nature, feasible validation, and inherent variability. In a previous publication we advocated that engineering model assessment methodologies of verification, validation and uncertainty quantification (VVUQ) could be used to improve credibility of models (Pathmanathan and Gray, 2013). However, while verification and uncertainty quantification methods are certainly relevant to CEP model assessment, best practices and quantitative methods in the engineering literature regarding validation appear less relevant to CEP models and other physiological models, due in part to the unique challenges in obtaining data for validation of physiological models. In general, the types of evidence supporting the credibility of physiological models will likely be very different to that for engineering models. Therefore, this paper is motivated by the need for a clear understanding of potential credibility evidence for CEP models, which can guide future efforts toward systematic approaches for credibility assessment/justification which are relevant to physiological models.

We specifically highlighted validation of general-purpose CEP models not performed for any prescribed COU, and defined this as “general validation” evidence. As discussed in section Why Trust a Computational Model? the ever-increasing complexity of CEP cell models means that there are almost unlimited possibilities for such evaluation, and there is a large and ever-growing body of general validation results in the CEP modeling literature—in particular regarding cell models. Note that in this review we described several examples of general validation but we did not discuss the “quality” of any general validation results. For example, we avoided subjective statements such as “validation results showed good agreement between model and experiment.” This is because the level of agreement needed between model and experiment is determined by the COU, and when no COU is specified, a statement that a model shows “good agreement” without any context could potentially lead to inappropriate use of a model. In general, while general validation can provide important preliminary information about a computational model, it may not be advisable to convert general validation results into binary “good”/“bad” or “acceptable”/“unacceptable” statements. However, when a COU of a model is chosen, previous general validation results can certainly be (re-)evaluated to determine how supportive they are of the model in the COU. This will likely require assessment of both the level of agreement between model and simulation, and also the relevance or “applicability” of the validation conditions to the COU; discussed in detail in Pathmanathan et al. (2017).

Currently, general validation results for cell models that are published in the literature are not collected, curated, or made available in one place. One resource that could potentially be useful for the cardiac modeling community is a resource on credibility evidence for cell models. The CEP modeling community already leads the way in model sharing and reproduction through the CellML repository and related software (Lloyd et al., 2008). The CellML language is a XML-based language for defining CEP cell (and other) models, allowing models to be defined unambiguously and easily shared, and the CellML repository serves as the starting point when using a cell model published in the literature. However, the repository does not include information regarding model validation results or other credibility evidence, and there is no way to easily look up such information. A sister repository containing model credibility evidence could therefore be useful to CEP model developers/users when deciding on which cell model to use for a particular COU. Examples of information that could be stored in such as repository include which emergent phenomena the model reproduces (and does not reproduce), and general validation results under a wide range of precisely prescribed protocols. One resource that provides a path toward such a repository is the Cardiac Electrophysiology Web Lab (Cooper et al., 2016). This is an online tool for easy comparison of multiple CellML-defined cell models under a wide range of protocols (which required the development of an XML-based language for specifying protocols Cooper et al., 2011). Being able to easily compare models is important because even models of the same species and heart region can behave quite differently; (see e.g., Cherry and Fenton, 2007). While the Web Lab does not currently provide explicit comparison to experimental data, it already serves as a potential tool for identifying which phenomena models can reproduce, and one can imagine an extension in which experimental data (from a wide range of sources and with full details on experimental conditions and protocols) are also included and comparison to model predictions are provided, both visually for qualitative comparison and perhaps quantitatively with appropriate validation metrics. In fact, inclusion of experimental data is one of the future plans of the Web Lab developers (personal communications). As stated above, we believe such results should not be converted into binary good/bad or acceptable/unacceptable judgements, or used to rank models. Instead, such a repository would serve as a rich resource by providing information needed for selecting between competing models for a particular COU, as well as providing validation results that could serve as a starting point for justification of model credibility for the COU. Moreover, if users were able to upload models and automatically run all protocols (already possible in Web Lab) and then compare against the experimental data, this would be a powerful tool for validation of *modified* cell models (examples of which were provided in section Organ-Level Models), i.e., for comprehensive “re-validation.” Note that we are *not* stating that an altered model should only be used if it “passes all validation tests.” Indeed, for many COUs, a model not reproducing given phenomena could be argued to be acceptable given the COU. The point is that trust in cardiac models can be improved by collection of evidence, glass box evaluation, and explicit justification that the model is sufficiently credible for the COU despite its limitations.

It can be difficult to determine whether results presented in publications are calibration or validation results, as we mentioned in section Credibility of CEP Models at Different Spatial Scales. Specifically, while figures may be provided in which simulation and experimental results can be visually (and qualitatively) compared, it is often unclear whether any model parameters were tuned, optimized or tweaked to obtain the agreement with the experimental data. When that is the case, the results are calibration results, which is fundamentally weaker credibility evidence than validation of the *completed* model. Therefore, ideally calibration and validation results should be presented separately. While we believe that the examples of validation discussed in this paper are genuine validation results, it is certainly possible that some are actually calibration results. We also mentioned how simulation studies using CEP models are often performed in which no validation results or discussion of model credibility is presented. Such studies essentially implicitly rely—not unreasonably—on the maturity of the field and the various sources of historical evidence that we discussed in sections Ion Channel Models, Cell Models, and Organ-Level Models. The problem with this approach is it can contribute to a lack of clarity in the literature about the trustworthiness of CEP models, which can potentially lead to overconfidence in CEP models by non-experts who are unfamiliar with model subtleties (see initial discussion in Gong et al., 2017) as well as under-confidence in simulation-based conclusions by those who are skeptical of computational models in medicine. Such skepticism may be one of the biggest hurdles that needs to be overcome for computational models to achieve their potential in medical applications. These issues could be addressed by a clear and explicit presentation of the rationales for credibility of models used in simulation studies, referring as appropriate to the different sources of credibility evidence that support the use of the model for the COU as shown in Table 1 (and/or appealing to the idea of models as representations, as discussed in section Why Trust a Computational Model?, when appropriate). As stated above, one aim is to argue that the model is sufficiently credible for the COU, despite model limitations. While there is no standardized method for determining what constitutes “sufficient credibility,” the risk-informed strategy of (NASA, 2009; ASME, 2017) provides one method. The basic idea is that the credibility that needs to be demonstrated for a model should be related to the risk associated with incorrect predictions. Two factors are used to determine model risk. The first is model *influence*, which is the extent to which the model predictions will influence the decision to be made or conclusions of the study, compared to other sources of information. The second is the *consequence* of incorrect decisions. For example, if a model is proposed to be used as the sole source of information in a safety-critical clinical decision, both influence and consequence are high, and the overall risk will be considered to be very high. Therefore, high credibility will be required of the model. In simulation studies, influence will often be high but consequence may be judged to be low, and overall risk may also be judged to be low, which means the credibility requirements are lower. Ultimately, we believe that routine and explicit justification of credibility will enable CEP models to have even greater impact in cardiac EP research, and facilitate their passage into clinical applications.

## Disclaimer

The mention of commercial products, their sources, or their use in connection with material reported herein is not to be construed as either an actual or implied endorsement of such products by the Department of Health and Human Services.

## Author contributions

PP: Devised the paper, performed review of literature, and wrote the paper; RG: Provided feedback and edits on all aspects of paper.

### Conflict of interest statement

The authors declare that the research was conducted in the absence of any commercial or financial relationships that could be construed as a potential conflict of interest.
